# Somatics and phenomenological psychopathology: a mental health proposal

**DOI:** 10.1007/s11017-023-09618-2

**Published:** 2023-05-25

**Authors:** Camilo Sánchez Sánchez

**Affiliations:** Poznan University of Medical Sciences, Doctoral School, Poznan, Poland

**Keywords:** Somatics, Phenomenology, Psychopathology, Bodily Movement, Lived Time

## Abstract

This work begins with a brief review – from the *physical education* movement that began in ancient Greece and is deeply rooted in 19th century Europe, to the *somatics* movement alive today. The review captures primary historical and conceptual references, relevant to the therapeutic-embodied exploratory work. Then, G. Stanghellini’s mental health care model [2] is reviewed. This model is considered within reflexive self-awareness and spoken dialogue: the main vehicles in relation with alterity and its consequences in the realm of psychotherapeutic encounter and intervention. This will highlight the individual’s bodily movement and inter-corporeal ‘proto-dialogue’ as a prior realm of therapeutic intervention. Next, a brief consideration of E. Strauss work [31] is presented. This paper’s hypothesis is that bodily qualitative dynamics highlighted by phenomenology are essential for an effective mental health therapeutic intervention. A ‘seed’ of a framework is proposed in this paper; this seed assesses some phenomenological assets of a positive conception of mental health, for which self-awareness education is key to develop skills such as *kinaesthetic intelligence* and *attunement* and to educate healthy persons who can promote edifying social relations and environments.

## Introduction

‘*Nature is a principle of motion and change. (…) We must therefore see that we understand what motion is; for if it were unknown, nature too would be unknown*’ [1, 200b: p. 12–14].

As the human person’s life, identity, and dignity are forged in the life-long strife with the unchosen – in oneself, others and the environmentment–﻿ positive mental health is a kind of achievement and a true social responsibility as a member of a social group. The approach proposed by G. Stanghellini [2] is fine-tuned to this reality, and offers the individual the possibility to re-synchronize through social dialectical interaction and affective co-regulation; it also allows the individual to exercise reflexive contrasting of position-taking on his lived experience. If so done, it leads to a healthy inter-subjective co-existence through irreconcilable differences.

Following Stanghellini’s exposition [2, p.1], to be human is to be in dialogue with alterity.^2^ Mental pathology results from a crisis in this dialogue and, therefore, mental health care is a dialogue-based method that re-enacts the former individual’s dialogue. *Dialogical spoken word* and *reflexive self-awareness* are important psychotherapeutic tools, which are prioritized in Stanghellini’s proposed model. He writes: “*A person cannot discern alterity within himself until he has made of himself an external reality by producing a text, and after reflecting upon it*” [2, p. 27]. That is, to encounter alterity (within), language and reflexive self-awareness are necessary. The individual’s dialogical relation with alterity – the fundamental ground for Stanghellini’s values-based, person-centered model for mental health – most probably emerges from the development of the *extended* or *narrative self* in early socialization, entailing reflexive self-consciousness, narrativity, introspection, self-transcendence, and self-conception [4, p. 551]. As stated by T. Fuchs, this extended narrative self – which entails conceptual competence regarding one’s sense of agency and self-story formulation – presupposes pre-reflexive self-awareness as the basic individual sphere of first personal givenness of experience [4, p. 550]. This basic individual sphere is that through which the basic form of selfhood or *the* *minimal self* is constituted, also called *mineness* or *Ipseity*, scaffolding the extended narrative self. The present work questions Stanghellini’s phenomenological proposal on mental health as it is conditioned to ‘spoken word’ and reflexivity [2], and instead suggests that mental health research should prioritize the therapeutic encounter, understood as the pre-reflectively embodied ‘proto-dialogue’ experienced between patient and clinician, rather than the spoken-word reflexive dialogue.

Accordingly, this work sketches a conceptual proposal from a phenomenological ground, focusing on bodily movement as a fundamental therapeutic asset for mental health research. It is developed in four parts, the first is a brief consideration of some relevant ideas proposed in the Somatics realm, the second is a brief critical consideration of Stanghellini’s proposal [2], specifically in regard to the individual’s relation with alterity, the third is a brief elaboration on E. Strauss’ work [6, 30]. Finally, the fourth part elaborates an intuition to frame the proposed work.

## Historical background

### Physical culture and harmonic gymnastics

The *physical culture*^3^ of the 19th century is considered by some authors (e.g., K. Mullan [8, p. 16]) to be the antecedent of *somatics.*^4^ Within the former movement, gymnastics is referred to any exercise, approach, or system of personal or physical cultivation. Stemming from this physical culture movement, *Harmonic gymnastics* arose as an experiential practice (not acrobatics) for self-cultivation, growth, and education, which incorporated movement sequences and techniques as a versatile and embodied therapeutic approach to movement. Some of the main contributors and pioneers of the upcoming movements were clearly influenced by Harmonic gymnastics – e.g., Genevieve Stebbins (1857–1934). Stebbins was originally an actress educated in Francois Delsarte’s system^5^ as a performer and dancer. Based on Delsarte’s expression system and influenced by Swedish Ling Gymnastics,^6^ Stebbins developed her own embodied movement methodology [9–11].

Along with Genevieve Stebbins came Hade Kallmeyer, Elsa Gindler (1885–1961), Mabel Todd (1880–1956) and numerous other contributors, whose work shared two common methodological principles: (i) they developed from bodily movement and resulted in a therapeutic effect, and (ii) they are sustained by an organic and integrated conception of health. These two conditions will be integrated in the concluding proposal.

Since the term ‘somatics’ has a phenomenological heritage, it is important to clarify its meaning. As Thomas Hanna (1928–1990) and Don Hanlon Johnson came from a philosophical background (and they were the individuals responsible for coining the term), they referred to Husserl’s use of the term *somatology* [12, p. 18] as a methodical inquiry of the body, complementary to the one done by biological and medical science, in that it addresses the body through the phenomenological methodology (i.e., from the experience lived by animated organisms), unveiling it as a dynamically structured type of experience.

Stebbins stated: *“[T]rue relaxation would mean a complete resignation of the body to the law of gravity, the mind to nature, and the entire energy transferred to a deep dynamic breathing […] To transfer energy by voluntary action and involuntary reaction produces the necessary equilibrium for the renewal of strength (1892: 77–78)*” [8, p. 22]. It is thus evident that body and movement were fundamental in the techniques she practiced (i.e., dynamic breathing and being aware of the sense of weight by releasing tension to gravity), and, as part of them, how the body’s dynamics are structured through antagonistic and complementary processes (i.e., equilibrium between *voluntary action* and *involuntary reaction*). According to Stebbins, from the methodical exercise of the techniques, a new sense of self emerges as a therapeutic resultant.

The balance between antagonistic and complementary processes is also central to Mabel Todd’s work, partially as a result of her training in Delsarte’s gymnastics at Emerson College. Part of this training included exercising *Harmonic poise*, which consisted in exercising one body part to function harmoniously with the whole, which entailed the *Law of Opposition*: “*different parts of the body aid each other in the attainment of any desired end*” [8, pp. 24–25]. This law of opposition was illustrated by the process of a child learning to walk, as a harmonious balance between opposing movements and set of muscles which, through exercise, result in the harmonious and effective balance of action – walking.

### Somatics

In Don Hanlon Johnson’s book *Bone, Breath and Gesture* [13], many of the somatic movement contributors mention that they either took physical culture classes or were trained as physical culture educators, which accords with Johnson’s and Mullan’s [7] claim that somatic work emerged from the physical culture movement in 19th century Europe and North America. Also following Mullan [7, p. 32], the term *physical culture* incorporated bodily education practices from diverse lineages beginning in the 1800s and was later replaced by the term *physical education*.

Mullan writes further: “*Physical culture systems were arranged in four branches based on Ancient Greek categories: Military, Medical, Pedagogical and Aesthetic Gymnastics*” [7, p. 32]. This fragment illustrates an interesting aspect of the physical culture education project, namely that these bodily exercises were used effectively for the defense, reestablishment, qualification and embellishment of *animated bodily life*,^7^ correspondingly. Paralleling this aspect of physical culture, somatics also produces results and is applied for those purposes, encouraging the use of individual resources for personal growth as a way to reestablish health. As somatics has developed through the exploratory and research work of bodily function, it addresses the lived experience of individual organisms. In Mullan’s words: “*The principles of somatics have developed out of multi-disciplinary theories emerging from direct experiential explorations of the body, breath, and states of being. Somatic disciplines are forms of complementary medicine often referred to as body therapies, or even as the ‘intuitive restoration of self’*” [7, p. 5].

Taking into account the philosophical-phenomenological heritage of somatics, and following Mullan’s presentation [7], I claim that through somatic work, individuals develop body-awareness that helps to reestablish health [7, p. 8], further suggesting that through the development and active work on body awareness, protective processes are activated. To substantiate the point, Mullan states: “*When needed[,] pain or physical malfunctioning signals our body as warning signs meant to inhibit action. In order to figure out why our body might be malfunctioning; somatic awareness activities allow the individual to access feelings or sensations from the body to develop a sense of personal movement habits and behavior*” [7, p. 8]. In other words, pain and sensations of discomfort outline a structure of possible action in certain moments, and through somatic work one gets to know oneself from the perspective of available behavioral possibilities.

Through its different pioneers and contributors, somatic work has gathered three common assumptions: (i) the action of directing attention to a body part immediately affects it, (ii) the body-awareness (of kinetic-kinaesthetic-tactile-proprioceptive change) influences physiological processes, (iii) the body-awareness provides health benefits [7, p. 72]. These assumptions could be loosely condensed to the effects of body-awareness (i.e., embodied change of conditions, including physiological), which opens up the possibilities for health benefit.

Following Mullan’s presentation [7, p. 11], Thomas Hanna elaborated the core of somatic work’s assumptions by considering the process of how focusing attention on bodily sensations activates certain brain areas that enact a change in sensory-motor coupling. He writes: “*Somatics involves experiencing the mechanics of movement and ‘cybernetics of coordination’. That is, through educated experience the body learns as a system how to improve upon its own movement patterns.*” [7, p. 11]. According to this fragment, part of somatic work is to gain awareness of the individual pattern of embodied qualitative (i.e., kinetic-kinaesthetic-tactile-proprioceptive) dynamics in one’s own movement, and of how this dynamic pattern opens up for transformation and improvement.

These dynamic patterns can be transformed by practicing new associations or conditioned patterns, and this capacity to guide one’s own transformations is referred to as *kinaesthetic intelligence.* Mullen writes:

Sherrington also further defined coordination in which reflex actions over time can be conditioned internally to a higher level of integrated movement, thus increasing kinaesthetic intelligence within the proprioceptive system through the practice of acquiring new motor skills. Increasing kinaesthetic intelligence is central to many somatic practices such as the Alexander technique [7, p. 62].

Considering Mullan’s statement, awareness is a necessary condition for the transformative process in somatic work and for kinaesthetic intelligence, as awareness refines and *clarifies* the individual landscape of behavioral possibilities, which by itself facilitates the transformation of individual dynamics. In her own terms: “*The most advanced movers, dancers, martial artists, and athletes are those who have heightened skills in movement awareness, otherwise known as kinaesthetic intelligence. Developing sensory awareness is a key component of somatic work as it is a building block from which greater movement capacities will grow.*” [7, p. 76]. According to this fragment, the ‘elite movers’ develop high skills in movement-awareness, enabling themselves with progressive movement capacities; these skills are known as kinaesthetic intelligence. But how are these *skills in movement awareness* to be understood? Certainly, it does not only entail an awareness of the moving body qualitative dynamics, but also the practical knowledge of this embodied dynamics that ultimately results in a higher transformative capacity (or in a generally higher level of behavioral efficiency) regarding somatic work.

In synthesis, awareness of one’s embodied qualitative dynamics is a necessary condition for kinaesthetic intelligence [7, p. 62] in somatic work. The practical knowledge of these dynamics is kinaesthetic intelligence [7, p. 76]. Since this type of intelligence is from the first-person perspective, the practical knowledge is of one’s own individual embodied qualitative dynamics.^8^

### Somatic work and transformative experience

According to the *International Somatic Movement Education and Therapy Association* (ISMETA^9^), the main goal of somatic work is to *enhance human processes of psychophysical awareness and functioning through movement learning*. And according to ISMETA, its main beneficial conditions are to:

- Focus on the body, both as an objective physical process and as a subjective process of lived consciousness.
- Refine perceptual, kinaesthetic, proprioceptive, and interoceptive sensitivity that supports homeostasis and self-regulation.
- Recognize habitual patterns of perceptual, postural, and movement interaction with one’s environment.
- Improve movement coordination that supports structural, functional, and expressive integration.
- Experience an embodied sense of vitality and extended capacities for living.

These goals and benefits are gained through the practical discipline that is somatic work, which includes various hands-on *body work* and *body awareness* approaches [15] designed to deeply change one’s habitual individual embodied patterns and inter-corporeal life as a whole [7, p. 18]. But *which habitual embodied patterns are the ones referred to here?* As a working hypothesis (1), an *individual habitual pattern of bodily qualitative dynamics*^10^ is posited, which structures organically the animated experience.

According to Mullen, “*if somatic practices change how a kinaesthetic system functions, then the correlative field of lived experience will be altered as well*” [7, pp. 20–21]. As Mullan’s statement presupposes, it is clear that if kinesthesia is a central ‘building block’ of the experience of an animated organism, and if the kinaesthetic dynamics is altered, then the experience of the animated organism will also get altered. As was considered above, kinaesthetic intelligence is a necessary condition for the transformation resulting from somatic work. That is, practical knowledge of the bodily qualitative dynamics is necessary for this transformation. This practical knowledge can be gained through somatic work as it’s can transform the bodily qualitative dynamics in its temporal unfolding, enabling the individual to withhold unhealthy habitual patterns, freeing the individual for healthier possibilities of behavior in the landscape of the individual’s animated experience in the present moment.

As Michael Murphy states: “*today we have strong evidence that any aspect of bodily functioning, once brought to awareness, can deliberately be altered to some extent for healing or the development of new abilities… therapy or healing derives from fundamental transformations of experience and the cultivation of new capacities*” [17, pp. 88, 386]. This fragment refers to the present moment of transformation, when the individual withholds the habitual unfolding of her/his embodied dynamics – as a consequence of the awareness – and thus opens up to the landscape of possibilities available for these dynamics to continue unfolding through another bodily way. The more an individual gets efficient in this transformation process, the more kinaesthetically intelligent he becomes, and is more capable of transformation by developing, educating and, ultimately, healing himself. This process is broadly referred by Yvon Jolly in these terms: “*This discipline* [Somatics] *is interested in the living body’s subjectively experienced capacity for self-education.*”

[7, p. 23]

Returning to kinaesthetic intelligence, certainly awareness is an aspect of this intelligence, but there is also another aspect of knowing *when and how* it is appropriate to modulate the embodied qualitative dynamics^11^ for an efficient intervention that leads to change and transformation; be it in the individual’s own sphere or on another individual’s sphere (think of the therapeutic health care relation). In any case, as long as the intervention develops an ability and/or reestablishes health, it presupposes an aesthetic component of feeling when and how it is appropriate to modulate this dynamic. Mental health care practitioners (including somatics contributors) have discussed to this broad aspect of the transformation experience. For example, Ninoska Gómez states that somatics is both an art and science in that practitioners combine scientific knowledge with aesthetic responses of the body, conveying ‘*intuitive aspects difficult to formulate into words*’ [7, p. 23].

Another aspect highlighted by Somatics contributors regards how this body of knowledge was developed and can continue being developed by exploratory work. Exploratory work emphasizes creativity for the development of more efficient therapeutic approaches on the one hand, and on the other, the scientific ignorance of the animated body as a sense-making source. Mirka Knaster adds on the goal of somatic work: ‘*While they are all different ways of working with the body, the healing process remains the same: What was static or blocked gets moving again; where there was separation or fragmentation, there is now unity and wholeness*’ [7, pp. 26–27].

This statement on the one same goal sought through different methodologies is intuitive and enlightening, as it points out phenomenon present when mental health is affected: *stillness* and *isolation*. These features can be, directly and literally, bodily and experimental (i.e., individual does not move nor interact). Moreover, they can be intuited as characteristic of the individual psychic condition, viewed through the hypothesized habitual pattern of embodied qualitative dynamics instantiated in movement dynamics. According to somatic practitioners, through their different methodologies, the former condition is surpassed to regain a healthy psychic condition.

Deane Juhan offers a principle of somatic work which begins to substantiate the claim that through somatic work an individual gets to discover/know himself: ‘.*if you cannot sense it, you cannot move it… the more you move it, the more you sense it*’ [7 p. 82]. This principle integrates the abilities displayed in *conscious movement*; through movement exercises (mainly kinaesthetic-proprioceptive-tactile memory and imagination), the individual gets the bodily *feel* of certain qualitative dynamics. In Juhan’s own words: ‘*Feeling states, the clear sensory memories of doing are the things that accurately guide our muscular actions, not analysis and conscious sequencing of steps*’ [7, p. 82]. According to Juhan’s statement, kinaesthetic-proprioceptive-tactile memory and imagination guide kinaesthetic intelligence in the moment of present transformation; this type of intelligence and resulting transformations are not (directly) results of reflexive awareness^12^ and conceptual content.^13^

Mullan contributes on how kinaesthetic intelligence is guided: “*Our capacity for developing more efficient movement habits is a result of paying attention to sensations from the body and consciously making the effort to reprogram our sensory motor system until we get a feel for it*” [7, p. 82]. She adds the conscious effort as a force that finds its bodily way through the individual’s qualitative dynamics, and then looks to reinforce the bodily way instantiated, with the aid of kinaesthetic-proprioceptive-tactile memory.

As part of this paper’s initial working hypothesis, I claim that this individual habitual pattern of embodied qualitative dynamics structures the individual experience, including unhealthy and harmful experiences. Some somatic practitioners and contributors have proposed that the reinforcement of habitual patterns of these qualitative dynamics can affect health. Mullan adds, quoting Juhan:

Repeatedly triggered instinctual muscular reflexes and reactions to daily stresses create habitual muscular contractions, chronic tension and bodily pains. Stress or emotional strains can trigger the trauma reflex, a ‘reaction of the sensory-motor system’ in which muscles flinch and repeatedly cringe into ‘stuck’ positions leading to postural disorders (Somatics 81) [7, p. 84].

As stressed in this fragment, an individual could reinforce certain patterns of embodied qualitative dynamics that structure certain sense-making experience, which could become a loop and/or corrupt the individual’s capacities. For example, in the case of trauma, certain aversive experiences could prompt a pattern of qualitative dynamics to unfold (such as global patterns of muscle contraction), initiating an auto-referential recursive sense-making experience that tends to reinforce itself (structuring closed postures with anxiety prompting negative valence ruminations) and limit and/or erode the individual’s capacities. A similar take based on habitual patterns of muscle contraction tone is proposed by Gerda Alexander: “*stating that reflexive* [Reflex-like] *habits are a result of ‘tonus adaptation’ related to certain emotional states*” [7, p. 85].

This somatics embodied approach to health, and to mental health in particular, has as one of its pillars the concept of *habit*, which is conceived as a reinforced, reflex-like, neuro-physiologically determined, sensory-motor coupling. Due to this notion, Hanna refers to its unconscious-involuntary nature as *sensory-motor amnesia* (SMA):

Even though habits become ingrained in our body in a reflexive, unconscious manner, ‘because SMA is a learned adaptive response, it can be unlearned’ (Hanna Somatics xiii).… Somatic work is therefore a form of education in which a client becomes able to consciously alter their own experience as a result of increased awareness [7, p.85].

As claimed in the fragment, it is conceived that habits can be transformed through conscious movement practical exercises, guided by kinaesthetic-proprioceptive-tactile awareness, imagination, and memory. The topic of habits is definitively one that deserves further research and study, due to its wide relevance and application in different academic and corporative fields. Nonetheless, this topic will not be addressed here due to space limitations.

Lastly, is important to highlight two final aspects of the somatics approach to mental health. First, the individual affected is treated as an *agent*, rather than as a *patient*. The somatic work addresses the health issues where the transformation or health reestablishment process is a pedagogical and re-educational one and is partially and consciously carried by the individual. Second, and derived from the first, as the individual is implicated as agent, a main responsibility in her/his health reestablishment process is on him, looking to integrate him as part of the solution rather than excluding him as part of the problem. This, of course, can be done as long as the individual is provided with professional training and guidance along with a methodology and quality-time of professional practice. If so, then this unique therapeutic and pedagogical process can be instantiated.^14^

This paper’s concluding proposal is founded on a conception of the human being that encompasses its animate and sense-making nature. The following is a synthetical presentation of this philosophical anthropology grounded in Stanghellini’s model.

## Philosophical anthropology

Stanghellini’s main aim is to answer three ontological broad questions: (i) “*What is a human being?,”* (ii) *“What is mental pathology?*,” and (iii) “*What is mental health care?”* [2]. His answer was mentioned above – he develops a person-centered, dialectical model, from which a comprehension of mental disorders and a values-based therapeutic approach is derived.

Accordingly, he proposes a distinction between *selfhood* and *personhood*:

The phenomenological notion of selfhood serves to explore the fact that we live our conscious life in the first- person perspective, as an embodied, self-present, single, temporally persistent, and demarcated being, who is the subject of his perceptions, feelings, thoughts, volitions, and actions. This basic form of self- experience is implicitly, pre- reflexively, and non- observationally manifest. The notion of personhood is markedly more comprehensive than the notion of selfhood. ‘Personhood’ helps to clarify the ways pre- reflective self- awareness is structured as an embodied and situated experience inextricably entangled with an experience of a basic otherness [2, p. 22].

According to the distinction, both selfhood and personhood are types of self-awareness experience – the former refers to implicit and pre-reflexive self-awareness which is observationally non-dependent, and the latter refers to *selfhood* situationally structured by the experience of another. This distinction builds upon Stanghellini’s conception of the human person defined by the structuring experience of another, and conceived as discursive and dialogical. He continues:

…In brief: we are a dialogue with alterity. We encounter alterity in two main domains of our life: in ourselves, and in the external world. In the first case alterity is in the involuntary dimension of ourselves, our un- chosen ‘character’, including needs, desires, emotions, and habits. In the external world, alterity is encountered in the challenging otherness of the events and in the meetings with other persons that constellate our life [2, p. 1].

The dialogical relation with oneself entails a necessary distinction in oneself-ness, one sense being *alterity* which embodies our involuntary nature (reactions, habits, moods, etc.), but is also sensed in what is voluntary to others and alien to oneself. Then, there is an alien nature in oneself, also sensed in others – i.e., a sense of ‘otherness’ in others and in oneself. The former is tautological, but the latter implies that, if the relation is towards a sense of ‘otherness’ (within oneself), then there is a sense of ‘sameness’ from which one relates to.

This is achieved through dialogue. Stanghellini writes: “*dialogue is a kind of ‘experience’: it is not merely a verbal exchange, an exchange of information; rather, dialogue lets something happen. What emerges in dialogue is neither mine nor yours and hence transcends the interlocutors’ subjective opinions*” [2, p. 9]. This fragment highlights that through dialogue a real and reciprocal ‘epistemological sharpening’ of the dialoguing parties can be accomplished, and even more, it can also point to the sense which constitutes inter-corporeal shared dynamics in dialogue, as a ‘proto-dialogue’ or *proto-conversation* [2, p. 20] scaffolding the linguistic interaction. Stanghellini then states: “*Dialogue is the main function of language*,” as it is through the former that alterity is encountered [2 p. 10]. That is, alterity is encountered through the dialogical unfolding of inter-subjective and the use of spoken language. He continues characterizing human nature through the implications of alterity, pointing out that the characteristically human inner dialogue with alterity implies a *continuous process of becoming*, through overcoming the immersion in mere now-moments via the ‘*way to the time of narration*’ [2, p. 10].

This is enlightening and accurate, reflective of a Heraclitean ever-changing human nature (i.e., one is never identical to / passed oneself). Classically understood, paradigmatic examples of this definition are *movement* and *time*, which is not surprising as they are the main elements of this proposal and necessary assumptions (along with *space*) of the concepts of change and transformation / becoming. Additionally, as alterity entails awareness of the individually unchosen (within oneself), it leads to the constitution of narratives with a coping function. This self-awareness is reflexive or pre-reflexive depending on its features and its preservation in psychopathological cases [20, chap. 3]. Nevertheless, insofar as this self-awareness comprises narrative skills and a corresponding temporal experience (‘time of narration’), it is an object-like or reflexive self-awareness. Taking into account that dialogue serves understanding, Stanghellini seems inclined to the following Buberian interpretation: “*According to Buber, the fullblown ‘I’ only emerges once one perceives oneself as a You, when interpersonal dialogue turns into self- centered inner dialogue.*” [2, p. 21]. As stated, there is a constitution of oneself only when one perceives oneself as a ‘you’ (i.e., when one is reflexively self-aware). Assuming that a healthy dialogical relation with alterity results in the constitution of oneself, then this dialogical relation entails a reflexive self-awareness.

Furthermore, the encounter with alterity (within oneself) not only entails a reflexive self-awareness but also recognizes language as vehicle. The latter can be derived from the above. This is more clearly stated in Stanghellini’s earlier work. He writes, thinking with Paul Ricoeur:

Alterity comes into sight, materializes in the pleats of the world/text I have produced. By unfolding the pleats of the text in front of me, I get a panoramic view of how the parts of the text are articulated…A person cannot discern alterity within himself until he has made of himself an external reality by producing a text, and after reflecting upon it. Personal narratives are the result of the integration of alterity, revealed by the distantiation and autonomization of the text; in the discourse of the self (Ricoeur 1990). To note, the essential question is not to recover behind the text the lost intention of the author; rather, to unfold ‘in front of the text, the ‘world’ which it opens up and discloses’ (Ricoeur 1981, 111) [25, p. 27].

In this latter fragment it is rather explicitly stated: To encounter alterity (within), language and reflexive self-awareness are necessary. Moreover, according to this fragment, the structure of the patient’s text uncovers the patient’s transcendental structures - deeply rooted in a discursive and linguistically articulated dynamics - as reflected in the patient’s narrative where alterity is encountered. Therefore, the individual’s dialogical relation with alterity – the fundamental ground for Stanghellini’s values-based, person-centered model – emerges from the development of the *extended* or *narrative self* in early socialization, entailing reflexive self-consciousness, narrativity, introspection, self-transcendence, and self-conception [4].

According to Fuchs, this extended self is grounded on Ipseity:

Despite this complex and dialectical structure, the extended self always remains based on pre-reflexive self-awareness: Only a being with the constant sense of mineness is ‘able to form concepts about herself, consider her own aims, ideals and aspirations as her own construct stories about herself, and plan and execute actions for which she will take responsibility’ (Gallagher and Zahavi 2005) [4, p. 551].

This extended narrative self which entails conceptual competence regarding the sense of agency and self-story formulation, presupposes pre-reflexive self-awareness as the individual basic sphere of first personal givennes of experience. This basic individual sphere is that through which a basic form of selfhood or *minimal self* is constituted, also called *mineness* or *Ipseity*; Ipseity scaffolds the extended narrative self [4, p. 550]. Within the mineness sphere or Ipseity, there is an assembly of dispositions, potentialities, and capacities anchored in the continuous temporal structure and inter-subjective common sense that constitute an individual embodied experience. This embodied experience entailed by pre-reflexive self-awareness is achieved by *passive synthesis* [26], which coordinates and constructs individual, embodied, and *animated*^15^ cognitive functions.

Bearing in mind that narratives function to cope with alterity (i.e., to make sense of one’s identity from the *otherness* in oneself as a founding factor in their identity in structuring their inter-subjective relations), Stanghellini introduces the function of the other in personal identity and mental health:

Our identity as a human person is a narrative identity that stems from the dialectics between what we are and the alterity that we encounter in our life…Ricoeur (1992) calls the dialogical process through which human existence develops the dialectic of sedimentation and innovation. Mental health is the equilibrate dialectic and proportion between sedimentation and innovation, that is, between the alterity that comes manifest through the encounter with one’s un- chosen, ‘involuntary’ disposition or with an event, and the capacity of the person to cope with, modulate, appropriate and make sense of them [2, p. 22].

From this fragment one can derive that alterity or the ‘other’ (‘you’) is a main structuring factor of oneself, one’s personal identity, and one’s mental health, each of which are essentially relational or social. And this constituted personal or narrative identity demands a reflexive self-awareness for a healthy dialectic relation (with alterity) to unfold. For Ricoeur, human development requires a healthy or balanced relation between *sedimentation* (or part of the individual’s otherness) and *innovation* (or the individual’s coping resources); it is through the latter that the individual manages to achieve a healthy and balanced development [2, pp. 121, 156].

The ‘you,’ other or alterity, structures itself as a counterpart, not only as the otherness but also as the different levels of reciprocal recognition, starting with the bodily posture and glance. Stanghellini writes: “. *the other’s look is constitutive of our selfhood and personhood. We need a ‘You’ who looks at us to form and maintain our basic self and personal identity. We need the recognition of a ‘You’ to become and remain an ‘I’*” [2, p. 19].

As stated by Stanghellini, the other’s look serves as a structuring feedback factor of one’s pre-reflexive situationally and inter-subjectively structured embodiment. The other’s embodiment as sensed (posture, movement, gesture, etc.) is potentially one’s way^16^ to deal with alterity in oneself and others (behavior, actions and events), including the reciprocal coping narratives put forward. This reciprocal and inter-subjective process of co-constitution, is not a once-and-for-all process, rather, it is a fluctuating dynamic of actualization, where the personal identity and the structured inter-subjective relation is frequently tested.

Stanghellini continues:

Neonates are innately prepared to link to their caregivers through imitation and affective attunement. This is the intersubjective matrix from which human life arises. This matrix is non- symbolic, non- verbal, procedural, non- propositional and not reflectively conscious (Ammaniti and Gallese, 2014; see Stern, 2004). It has a “protoconversational turn- taking structure” (Stern, 2004, p. 21) [2, p. 20].

This fluctuating co-constitutive dynamics and, therefore, the individual’s embodied structured manner of relating with others, ontogenetically dates back at least to the primal inter-subjective matrix referred by Stanghellini (i.e., an innately conditioned link to others based on two main skills: *imitation* and *affective attunement)*. This primal inter-subjective matrix is characterized as non-representational, non-propositional, non-conscious, non-reflexive, and procedural, embodying the sense conveyed in a non-linguistic manner. Accordingly, the full-blown inter-subjective dialogue has as its ‘seed’ these primal inter-subjective dynamics, and therefore, the sense conveyed through the latter is a primitive and radically embodied sense, conveyed through movement in a proto-conversation.

Two of the main sources of alterity are one’s body^17^ and the other, instantiated in all types of circumstances. Relative to one’s body, Stanghellini articulates\ a radical inherited antinomy:

My body is an ambiguous thing. It represents an intimate aspect of the challenge of alterity: ‘To the extent that the body as my own body constitutes one of the components of mineness, the most radical confrontation must place face- to- face two perspectives on the body— the body as mine, and the body as one body among others’ (Ricoeur, 1992, p. 132). My body is the most intimate part of my identity as a person, and yet it is also an integrated part of an anonymous nature that does not care about what I want and who I feel I am [2, p. 25].

One’s body inherits a radical antinomy as it instantiates *individuality* and (a corporeal basis of) *we-experience* [47], both of which can be evidenced at multiple levels. The former is observationally literal and the latter instantiates the non-individuality of sharing a common embodied phylogenetical history and nature.

According to Stanghellini, there are three main ways of “dissociated spontaneity*”* that instantiate alterity: drive, habit and emotions [2 pp. 30–52]. And, as a vital human factor, alterity has three main sources in the individual’s life: (one’s own) history, body, and world. From these, three core nucleus of values emerge: historical (e.g., family’s values), organic (e.g., sexual preferences), and social (e.g. rules and roles). These core dimensions to dissociated spontaneity, sources, and nucleus of values constitute the individual’s involuntary dimension, sedimenting dispositions and setting boundaries to freedom.

Regarding *drive*, it responds to two basic bodily experiences (i.e., need and desire). Stanghellini characterizes *habit* as a non-reflexive practical way of thinking (‘cogito’). Assuming the voluntary is equivalent to reflexive decision-making, habit is involuntary and opposes the ‘habitual’ and ‘reflexive’ self [2, pp. 36–37]. Based on this conception, habits serve as a great foundation of behavior, as they are embodied ways of procedural memory instantiated through activity and acquired through the individual’s history. However, habit is not merely limited to movement patterns and overt behavior, it is more powerful as it embodies whole ‘chunks’ of experience, and influences their temporal constitution and unfolding, evidenced through coping skills developed to deal with alterity (e.g., narratives). In Pierre Bordieu’s illustrating words on how habits function as an embodied phenomenon, it is as if one’s ‘*entire body is full of mute imperatives*’ [2, pp. 36–37]. This powerful aspect of habits is also expressed in social interaction, as the temporal structuring process of experience supposes continuous anticipation and retention of sensory feedback [27, p. 50]. Habits scaffold this process almost without the individual’s awareness, leading him through known cues of bodily kinetic dynamics to known bodily qualitative dynamics [14].

Stanghellini continues:“*My habit is what prescribes me with a limited set of expectations of self- with- other schemes of interaction*” [2, p. 37]. The statement carries some assumptions about the bodily: (i) observed patterns of bodily kinetic dynamics usually embody specific inter-subjective sense, (ii) felt patterns of bodily qualitative dynamics usually embody specific individual sense, (iii) there is an individual self-differentiating pattern of kinetic-kinaesthetic animated dynamics functioning from the individual history of embodied experience referred by (i) and (ii). As implicitly stated, these bodily assumptions are defined by the individual embodied history, which constitutes an inter-subjective pattern or ‘style’ of relation, as the individual’s ‘map’ of the self-with-other ‘land’ [29, p. 283].

Taking into account D. Stern’s [28] contribution, Stanghellini explains that habit is activated through a prototype form of self-with-other relation, which becomes a generalized and encoded association. And as this association is enriched with detailed variations, a habitual behavioural repertoire is achieved: whenever an attribute of the inter-subjective relation is instantiated, then a habitual response is activated, as an implicit procedural memory pattern of interaction. He writes:

*These schemes of being-with form \a coherent and persistent structure that consists in a form of practical, implicit Cogito that drives my body to behave in a given way under given circumstances. It is an endless invitation to repeat the same schemes of actions, perceptions, and interactions* [2, p. 37].

These habitual schemes of being-with or behavioural repertoires become an economical way of governing oneself (‘thinking’), which functions through an inter-subjective structure of common and repetitive behavioural pathways; again, as a shared and limited road-map of the inter-subjective ‘land’. Relative to the other constituent of the individual’s involuntary dimension (i.e., emotions), and based on the above, in emotional experience is manifested the dialectics of personhood and alterity. There is a challenge posed to one’s own identity through the dialectics of the voluntary and involuntary of what one is. This challenge is experienced on a daily basis, and *the task of continuously coping is what constitutes a person.* In other words, emotions are part of the alterity with which a person is continuously challenged, and the outcome is what constitutes the person as who one is.

Stanghellini refers to the temporal unfolding of affects as part of the dialectical dynamics of personhood by pointing out that top-down emotional constitutive processes (affects → moods) unfold pre-reflexively, but bottom-up processes (moods → affects) unfold reflexively. He writes:

This basic emotional tonality is a permanent, implicit protention [sic], or readiness to (re)act and be affected in a given way, and probably also to develop certain moods more than others… It is important to notice that all these transformations from affects to moods to character occur pre- reflectively and without a deliberate and thematic involvement of the person in the process, whereas the transformation of a mood into an affect involves reflection [2, p. 44].

A fundamental affective and inter-subjective aspect, where the dialectical dynamic of alterity is instantiated, is the reciprocal one-other’s recognition. There is a teleological agency in inter-corporeality motivated by a basic human desire for (other’s) *Recognition*. Stanghellini conceives the latter as a ‘we-experience’ for which reciprocal affective attunement is a necessary condition, discarding understanding as an identification of the other’s mental state and approval/consent as an ethical judgement or stance:

Recognition first and foremost presupposes attunement with the Other. It is a mode of being with the Other, a kind of intimacy with the Other, a modulation of the emotional field in- between myself and the Other. In an essay significantly entitled Making music together, Alfred Schutz (1976) explains that the experience of the ‘We’ that is at the foundation of all possible communication is a mutual tuning- in relationship, a sharing of the Other’s flux of experiences similar to that of two co- performers (let us say a soloist accompanied by a keyboard instrument) who have to execute a piece of music [2, p. 50].

He states that “we-experience” is a kind of inter-subjective experience, in which individuals reciprocally regulate affectively through the sharing of each one’s flux of experience (with each other). The question becomes: “*What do they share in that experience*?” – this question is necessary to make sense of the ‘*we.’* In agreement with Stanghellini, individuals in a “we-experience” share a common temporal experience or their experience becomes synchronized [2, p. 50]. An individual’s coordinated and embodied ‘rhythm,’^18^ or integrated temporal experience, is instantiated in bodily movement (e.g., in the coordinated dynamics of facial gestures, ocular movement, whole-body postural adjustments and movement of the extremities), serving as mediation for a reciprocal embodied affective tuning. According to Stanghellini, the face-to-face relation is what constitutes the common temporal experience, as the synchronization is achieved through the reciprocal and continual mirroring of each other. Lastly, it is pointed out that “we-experience” is inversely proportional to the individual’s awareness.

The above is a brief presentation of the main methodological elements of Stanghellini’s proposed model for mental health care: *Phenomenology, hermeneutics and psychodynamics*, or PHD [2, p. 117].

### The phenomenological, hermeneutical and psychodynamic model

Stanghellini’s mental health care proposal (PHD) is based on a dialectical conception of the person as the basis for the comprehension of mental pathology and a values-based practice. Based on this conception, he understands mental pathology as the outcome of a crisis in this dialogue. Therefore, mental health care is a methodology in which (through dialogue) the individual’s dialogue with alterity is restored or re-enacted. This methodology requires techniques and devices that respond to *logocentric* (‘logos’) and/or *anthropocentric* (‘pathos’) sides of the therapeutic dialogue. The logocentric side is the methodical unfolding of the individual’s life-world, which is the pre-noetically constituted structure of experience, through which the individual makes sense of himself, the other, and the surrounding environment; it looks to inter-subjectively re-build and preserve this fundamental phenomenological structure. The anthropocentric side is the fully-fleshed practical (clinician’s) disposition to be the other’s dialoguing partner; it is the technique of being able to attune and emotionally regulate the patient.

The latter is synthetized in the following fragment:

In values- based practice, value- pluralism and recognition are the basis for clinical practice. This statement reflects the ideal of modus vivendi that aims to find terms in which different forms of life can coexist, and learn how to live with irreconcilable value conflicts, rather than striving for consensus or agreement… A person’s symptom is the outcome of her need for self-interpretation with respect to her encounter with alterity, that is, with puzzling experiences [2, p. 65].

This values-based mental health care model has pluralism and recognition as the main postulates for clinical practice. The clinical practice derived from this model looks for a continuous inter-subjective interaction of irreconcilable value conflicts; it discards agreement or consensus as a goal or solution. And, a symptom is understood as the extreme outcome from the individuals encounter with alterity.

This person-centered understanding of mental disorders treats patients as agents already in their own therapeutic process, as they can take an active role shaping their symptom(s), course and outcome. This pathogenic trajectory for clinical application is synthetized in three steps [2, p. 66]: (i) a radical disproportion between alterity and the individual’s innovative coping resources, affecting the structure of his experience, (ii) the individual’s miscarried self-interpretation of his affected experience, (iii) the individual’s fixation on the psychopathological structure, affecting the person-alterity relation.

Bearing in mind Stanghellini’s conception of the human person, psychopathological phenomena are a natural and normative possibility (i.e., individual dialogue with alterity can lose balance and, therefore, affect the individual’s structure of experience and identity). Accordingly, this phenomenological therapeutic approach is based on dialectical relation with the other; it looks to re-enact or re-establish this human dialogue with alterity, which entails a reciprocal reflexive self-awareness. The seven principles founding this proposal are:

1. *The patient’s subjective experience is the main focus and point of departure of any clinical encounter.*
2. *Encourages the patient to reflect and express narratively his experience, looking to make it meaningful as a whole (meaning-organizer).*
3. *Supports the patient in making explicit his own horizon of meaning (values, beliefs, etc.), in which narrative is set.*
4. *Suggests the clinician making explicit his own understanding of the patient’s narrative (assumptions, personal experiences, beliefs).*
5. *Clinician makes explicit his own meaning horizon, relevant to the therapeutic purpose.*
6. *Clinician promotes reciprocal exchange of emotions and perspectives, and shared commitment to co-construct a narrative that integrates both original narratives.*
7. *Clinician tolerates diversity/conflict of values, beliefs, and facilitates co-existence when consensus is not possible* [2, p. 117].

These principles reveal two important features of the relation between clinician and patient: (i) the main vehicle is linguistic, (ii) the main structuring awareness is reflexive self-awareness. As stated, there are three academic approaches articulated by Stanghellini in his proposal: *Phenomenology, hermeneutics and psychodynamics* (PHD). The first is applied as a tool to discover the structure of the individual´s experience:

The main aim of this process is to rescue the logos of the phenomena in themselves, which is immanent in the intertwining of phenomena. But it also helps to recover the implicit (not necessarily rejected), automatic (not censored), forgotten (not forbidden) sources that make phenomena appear as they appear to the patient, his drives, emotions, and habitus — the three emblematic components of the obscure and dissociated spontaneity that make up the involuntary dimension in human existence [2, p. 118].

The phenomenological description of the patient’s experience, based on his reflexive narration and inter-corporeal interaction, helps the clinician to delineate an individual’s animated embodied and inter-corporeal ‘style’ [29, p. 283], instantiating drives, emotions and habits. This method also incorporates an intentional component by uncovering the patient’s animated structure(s) of experience which embeds the flux of his embodied dynamics. From the perspective of hermeneutics, one focuses on the way the patient ‘digests’ the change in his experience, as he fixates on it, trying to make sense of it. This interpretation window and the patient’s dealings with it have to be carefully handled in their unfolding, as the sense constituted (through them) is the focal point for therapeutic intervention. Stanghellini writes:

The patient, with her unique strengths and resources as well as her needs and difficulties, has an active role in shaping her symptoms, course, and outcome. Rescuing from the implicit the active role that the patient has in shaping her symptoms is the via regia that helps the patient recalibrate her dysfunctional, miscarried position- taking and, finally, to recover her sense of responsibility and agency [2, p. 118].

Part of the therapeutic goals of the PHD model are not only to re-adjust the individual’s position-taking on his affected experience, but to habilitate or re-habilitate the patient’s sense of agency and skills and, therefore, re-establish his human dignity as an accountable person.

Lastly, and third, the psychodynamic component is coherently integrated with the others by providing an individual history and context embodied in the patient’s alterity and renewable (coping) resources. The main assumptions integrated in the model are psychological continuity and determinism. Stanghellini writes:

The former assumes that all of any person’s psychological events (including those that look inconsistent) are lawful and potentially meaningful in a particular way for that person. The latter presumes that all psychological events have at least as one of their causes a psychological cause and can thereby be explained on a psychological basis [2, p. 118].

This fragment suggests that the whole of experience (or psychic life) in an individual is *cohesive*, *connected*, and *continuous*.

This model proposed by the author serves as a guideline for the development and application of particular therapeutic methodologies. The model proposes five developing steps: (i) ‘*unfolding of the phenomena of the life-world and its structure*’, (ii) ‘*rescuing the self-structure*’, (iii) ‘*narrating the transcendental origin of the life-world*’, (iv) ‘*clinician’s appropriation of the patient’s life-world*’ and (v) ‘*grasping the importance of the patient’s life-world*’ [2, pp. 118–121]. The first, *unfolding the phenomena of the life-world and its structure*, is a process mainly developed through the spoken word of the dialogue between the clinician and the patient, resulting in a written text describing the patient’s lived experience. Stanghellini writes: “The product of unfolding is a text that reflects the phenomenal world, the world as it appears to the subject of experience… it is important to note that this process of unfolding is profoundly rooted in hearing— or even better: listening and dialoguing— and in the power of the spoken Word” [2, pp. 118–119]. One of the goals of this unfoldment is to dialogically understand (‘logos’) how the psychopathologic lived phenomena is integrated to the whole of the individual’s experience or psychic life. Stanghellini continues:

Phenomenological psychopathology advocates the idea that the phenomena embedded in a given (normal or abnormal) form of existence are a meaningful whole… [the standard concept of ‘syndrome’ refers to a collection of symptoms clustered by being effect of the same neurobiological cause]… This alternative perspective holds that the manifold (abnormal) phenomena in a syndrome are meaningfully interconnected, that is, they form a structure… To have a phenomenological grasp on these phenomena is to grasp the structural nexus that lends coherence and continuity to them, because each phenomenon in a psychopathological structure carries traces of the underlying formal alterations of subjectivity [2, p. 118].

This fragment begins by stating Jasper’s take on the particular (i.e., an experienced phenomena is cohesive, connected, and continuous with the individual’s nearby temporally structured and lived experience); lived experience being a dynamical, embodied, sense-making, organic structure in continuous flux. The phenomenological work helps to unearth these structures in their constitutive dynamics and in their sense-making or self-constitutive patterns. For example, as an individual’s relation with alterity is altered due to its own fixed interpretation of his experiential changes, if she/he takes into consideration these fixed interpretations and changes, (most probably in response to an experienced vulnerability^19^ or trauma^20^), these changes (can) alter the individual’s identity dynamics, continually re-instantiating (loop-like) the altered lived experience or embodied qualitative dynamics, as an identity or self-constitutive statement.

Stanghellini points to the common individual structure, between the ‘life-world’ and the ‘self-structure:’ “This is an exploration of the implicit structures of experience, or into the structures of the self as the tacit and pre- reflexive conditions for the emergence of mental contents.” [2, pp. 119–120]. In other words, an individual structure that serves as the pre-reflexive condition of human experience is the individual pattern of embodied qualitative dynamics which constitutes it. As the experience gets altered, the embodied dynamics alters as well. But the individual structure of experience - one’s personal relation with alterity and the individual’s identity - (can) suffer a truly enhanced disturbance from the individual’s fixed interpretation of the initial change.

Stanghellini draws upon the genetic phenomenology to account for a temporally constituted phenomenon as the individual’s self-structure. Of the model’s two final steps, it is important to point out that embodiment serves as an equalizer in the therapeutic relation: *The clinician appropriates the patient’s life-world* *through the phenomenological approach*, as the former imagines how it would be living in the latter’s lived-world, and also *grasps the importance of it* by considering the unchosen sense that the patient’s symptoms have. Both of these require the patient to relate to the clinician as a ‘you’ (‘other’) and the clinician to make sense of the patient’s identity and symptoms through a specific embodied attunement, ‘as-if’ the clinician lived the patient’s experience [2, p. 121].

This individual self-structure (or pre-reflexive self-awareness realm) is temporally structured through synthetic processes derived from the flow of consciousness [27, p. 93]. And this flow of consciousness is also deeply rooted the display of self-movement [18, p. 28]. Thus, one initial finding of this paper’s intentional analysis is that bodily-movement and temporal experience entail the flow of consciousness in their constitutional processes. Accordingly, a brief consideration of E. Strauss’ proposal will be presented.

## Bodily movement and lived temporality

Erwin Strauss elaborates a rich, vast, scientific, and encompassing synthesis of some of schizophrenia’s psychopathological manifestations regarding time and space, for which he proposes a basic distinction between the *abstract* or *allocentric* and *concrete* or *egocentric*^21^ [30, p. 35]. As Strauss stresses (and phenomenology has pointed out), science has dismissed the subject itself by ignoring the experience-based or lived temporality and spatiality in the psychopathological realm. This is due to the conceptual framework promoted by science and has led to the misunderstanding of lived-structures and the progression of psychopathological manifestations, in particular with how they intertwine in syndromes.

One of Strauss’ main goals is to characterize thoroughly how experience is structured spatially and temporally, looking to empower the clinician to identify a patient’s disturbance independently of the patient’s communication skills (i.e., based on the behaviour and bodily movement^22^). The latter, although highly improbable, would require reciprocal and continuous feedback between philosophy and experimental science, specifically the health sciences, as this pre-reflexive self-awareness realm affects the whole experience of the living organism. Thus, a sound philosophical analysis would have to be integrated to the biomedical one, looking to scientifically understand the pathogenesis of many diseases and clinical conditions according to the living sense construed by the organism itself; here, all organic events remain coordinated by the self-made sense. The latter is identified by Strauss through the *Physiognomy* concept (i.e., animated experience is lived in terms of *tempting* or *threatening*, *resisting*, or *supporting)* [30, p. 77].

Regarding the physiognomic quality of psychopathological manifestations, temporal disturbances are understood as a *loss of the egocentric structure*. It is through this structure, which these patients seem to have lost, that the patient makes sense and moves to engage in interaction with others and the world. This movement-based interaction between the individual, other, and world is coordinated through anticipation of movement/behaviour and its consequences. The verbal reports of psychotic patients seemingly capture some of the felt sense when this “anticipation dynamics” is damaged (e.g., a patient claims ‘*Time has ceased to exist*’ [31, p. 93] or when E. Minkowski’s patient claimed ‘*There is nothing but immobility around me*’ [32, p. 99–100]). The flow between the individual, other, and world, which constitutes temporal experience or lived time and entails bodily movement, is rooted in the flow of consciousness, and the flow of consciousness influences the individual’s negotiation of their social being. This milieu of phenomena and issues must be researched through a methodology that makes them directly available.

Following Augustine and Bergson, Strauss distinguishes two concepts of time: *objective* and *subjective*, both very much resembling the distinction between *abstract* or *allocentric* and *concrete* or *egocentric*, correspondingly [31, p. 88]. The former alludes to a third-person conception of time, not only ‘suffering from metaphysical anemia’ [31, p. 89] but also from *epistemological neglect* or *amnesia*; and the latter alludes to a first-person conception of time [31, p. 83], which, as phenomenological psychiatrists like Minkowski have pointed out, alludes to a major grounding of mental health [33]. In principle, this phenomenologically transparent phenomenon (concrete or lived time) structures the animate organisms lived experience and their primal being alive.

The general purpose in much of Strauss’ work is to grasp the functioning of the whole temporal process and account for its deficiencies. In *Temporal Horizons*, he looks to articulate two conceptions of time – abstract and concrete – through the notion of ‘*Today.*’ This notion entails “world-events” on which an objective time and lived experience are defined; both are linked through the (individual’s) ‘*state of becoming*’ which requires a profile of *Where-to’s* and *Where-from’s* that track the agent’s (potential) movement [31, p. 92].

It is important to acknowledge the links between movement and temporality. The abstract conception of the latter entails regular or cyclical movement (e.g., from the planets, stars, or clock pieces), and its concrete conception entails deep intertwinement [18, p. 28], but needs further clarification. Consider that the notion of the *state of becoming*, although broad, frames an approach on mental health grounded on the movement capacity of organisms, which coordinates an individual’s happenings with environmental events in a seemingly complementary way, so that the relevant movement^23^ vectors could very much come from anywhere within the whole temporal continuum (i.e., either molecular-neural processes, phenomenologically pre-intentional ones, or existential goals, all serving constitutional aspects of subjective individuality). Strauss’ patient’s narratives are revealing:

Erwin W. Straus: I have been told that you have some unusual experience with time, is that so? Patient: It is true. I can’t get time straightened out. I don’t know, it is not a continuous sort of thing. (…) Well, it seems to me that there is no such thing as a yesterday or a tomorrow or a future (…). Time to me is like climbing stairs – literally – and that you reach a certain point and then you fall down (…) [31, p. 94].

The patient seems to be living a *stillness* or *stagnancy*, where the temporal flow of time has become replaced by a sort of goal-dependent change (‘climbing stairs’), and the goal is an impoverished one, reduced to a ‘way out.’ The basic circadian rhythm, although probably acknowledged by the patient, has been dismantled of its basic sense of paced ‘movement.’ And this basic sense of temporal experience, as a progression or ‘moving forward,’ not only is lost but seems to be susceptible of being ‘crumpled.’ The same patient, when asked about the usual experiences of ‘now’ and of others/objects movement around him, answers:

At this time I quit progressing…Motion doesn’t seem to have any significance. I know that my hand is moving but there is no such thing as its actually moving from one place to another and that it happens because I am making it [31, pp. 94–96].

The patient’s sense of temporal experience as a progression or ‘moving forward’ is disrupted or abandoned, suggesting a relevant role of motion and the sense of agency in temporal experience. Therefore, retention and protention dynamics are constitutive of every temporal experience and its sense of flow. When disturbed, it is no surprise the patient is deprived of the sense of movement including the one coming from an other’s or an object’s. This disturbance is also pointed out by Strauss as the ‘proper’ psychotic manifestation: *disarticulated temporal and spatial experience* [34; 33, p. 93]. The psychotic patients not only suffer a disturbed temporal experience; their spatial experience is accordingly disturbed. The patient experiences everything as out of their reach, their ‘here’ is severed from any ‘there,’ i.e., the physiognomy of their experience is of isolation.

The phenomenological variables elaborated here are conceptual tools, which not only have a potential impactful effect on mental health,^24^ but also can contribute to the health sciences (as the lived experience coordinates the whole functioning of the living organism). These variables lay the ground for social interaction and self-embedded structuring processes on which health is sustained.

In the following, a summarized intuition is proposed for future experimental research in Neuroscience, Mental Health, and Philosophy.

## Proposal: embodied movement and temporality

The importance of dialogical spoken word and reflexive self-awareness as instruments of the proposed [2] therapeutic intervention has been shown above. And, although they are fundamental for psychotherapy in general, in particular they emphasize the therapeutic focus on the dialogical, inevitably dismissing (as secondary) the prior ‘proto-dialogical’ landscape of the inter-subjective relation. In contrast, this paper’s developing proposal looks to invert priorities, prioritizing the ‘proto-dialogue’ as focus of the therapeutic intervention over the dialogical landscape. Of course, the reflexive realm will always be an important asset in any therapeutic intervention. In other words, what is being proposed is that the individual’s relation with alterity is primarily^25^ instantiated with one’s and the other’s bodily movement prior to the talking/dialoguing (i.e., the first encounter with otherness is through the embodied qualitative dynamics that builds up to the split-second reaction).

This proposal has two components:^26^*theoretical* and *therapeutic*. In this paper, only the theoretical will be addressed. Based on the above, the constitutional process of Ipseity and alterity is begun through the pre-reflexive self-awareness realm, mainly through the unfolding of individual movement and temporality patterns, and as the proposed work is seeking to develop a mental health proposal which looks to study these disturbances through embodied movement, this paper’s the main focus must be placed on this realm. Thus, an embodied proto-dialogical therapeutic work will be proposed below, which may also benefit the dialogical and reflexive aspects of therapeutic intervention.

This paper’s proposal is framed by the following hypothesis:

…we consider self-affection as an outcome of incessant differentiation and coalescing of affective moments (see also Rogozinski 2010). In the process of differentiation some of these moments acquire the potential to form an alterity. The structure of the self-shows itself in dynamics of bifurcations and fusions of affective moments. This pre-reflexive circular movement enables self-reflection by offering a fissure, which normally becomes rapidly sealed and submerged assuring the sense of self-coincidence. In schizophrenia, this fissure becomes congealed, allowing for alterization and a formation of obtrusive quasi-subjects [43, p. 9].

This hypothesis is based on identifying subjectivity’s structure as the individual’s dynamic relation with alterity; which further entails a continuous dynamic of differentiation and coalescing during affective moments. These dynamics instantiate in the pre-reflexive awareness realm, as Parnas and Stephensen’s state, but is not only through affectivity that this dynamic can take place. For example, reflexivity is a realm of conceptual content of experience, where the differentiation and coalescing with others continually takes place.^27^

Parnas and Stephensen’s hypothesis [43] are partially inspired by Bin Kimura [44] and Henry Ey [45], especially on Kimura’s notion of *áïda* or *in-between-ness* (i.e., a bi-directional co-constitutive relation of the individual, with the world and other). This relational concept is applied by Kimura to explain the individual’s constitution through a circular co-constitutional dynamic of differentiation and coalescing:

...the notion of ‘in-between’ is not to be understood as a static or spatial distance between two different things, but rather as a dynamic and generative movement that differentiates one thing from the other… it seems to be the dynamic nature of the ‘in-betweens’ that is disturbed in schizophrenia, in that, the otherness of subjectivity has become concretized or thickened [43, p. 10].

Kimura’s notion of in-between-ness illustrates a co-constitutive individual dynamics or *generative movement* with alterity, instantiated in the world and other. It is through these dynamics that the individual itself – its world and the relation with the other – is constituted. Mental disturbance is thus understood as a result from the unhealthy^28^ constitution of these relations. Stephensen and Parnas continue:

In other words, the precondition for being able to recognize oneself, on an implicit or immediate level, consists in the interplay of integration and differentiation. Alterity is thus a constitutive feature of subjectivity, and in schizophrenia this alterity remains exposed or non-integrated, and one becomes Other to oneself in a concrete manner [43, p. 10].

This proposal has focused on the pre-reflexive constitutive processes as the main psychopathological genetic realm in general. Differentiation and integration are the necessary conditions for the pre-reflexive individual constitution. As Stephensen and Parnas state: “[In Levinas’ words,] *‘The I is not a being that always remains the same, but is the being whose existing consists in identifying itself, in recovering its identity throughout all that happens to it. (Levinas 1979, 36’*” [43, p. 11]. The point is that the animated life is built - and lived-experience is sedimented - in the continuous challenge of alterity. This strive always has consequences: individual and social, damaging or re-establishing, weakening and/or empowering. This life-long encounter with alterity, from which individual identity is achieved and harnessed, entails a dynamic pattern of experience as a flow between two *ideal* poles: the *radical individuality* and *the “we-experience*.” These constituting poles embody the self (Ipseity) and otherness (Alterity), correspondingly. The former pole could be instantiated in the flinching reflex, similar to the startle reflex [14, p. 94], as it’s the embodied experience of a radical individual distinction from situated alterity (e.g., this reaction could be instantiated when someone opens the shower to a very cold water or is attacked). The latter pole could be instantiated in the lordosis reflex, as it is the embodied experience of a radical individual fusion with situated alterity (e.g., this reflex is instantiated in copulation). This latter pole is not necessarily understood as affective, rather, it refers to a general broad kind of experience in which individuality ‘dissolves’ into a social phenomenon. These reflexes are proposed as paradigmatic examples and constitutive prompters of the aforementioned embodied senses. However, there are other fitting examples.

There are phylogenetic and ontogenetic conditions defining individual sense-making routines (e.g., the use of affable gestures when presenting a disagreement with the social group, to probe the probable response of the latter) [46, p. 8]. These routines habitualize individual patterns of lived experience consisting of temporal sequences, constituting a sort of behavioural repertoire; some being due to the individual’s own history, some being species-specific, and some others being genre-based. These sense-making routines, as habitual embodied temporal sequences, unfold encompassed to the lived body – that is why there seems to be an individual temporal period or rhythm according to which these routines are displayed (e.g., these sense-making routines are instantiated in how often an individual looks or speaks to another, initiating a conversation). This temporal period or rhythm partially defines the *unfolding* of the individual’s experience and its constituted sense (e.g. two individuals face-to-face, mutually flirting or confronting as a threat). Depending on the temporal period or rhythm of anticipation of an individual’s experience, the sense constituted could vary significantly if the other inhibits a response for a temporal period in which the former’s anticipation and corresponding sense-making routines are frequently displayed.

Thinking with Fuchs and Pallagrosi, “*Hence, we can assume that distortions of the lived time are always involved in the core of psychic disturbances and give rise to different symptomatic manifestations*” [42, p. 297]. This observation points to the relevance of temporal consciousness in phenomena as delusions. The fragment supports this idea by postulating a temporal distortion in every mental health disturbance. In this proposal, mental health is conceived as a product of the temporal and passive synthesis constitution of consciousness, instantiated and enhanced through embodied movement and inter-corporeal shared dynamics as a balanced flow between the distinction and fusion experience. And this flow emerges from the dialectical constitution of Ipseity in the relation to the environment and others, entailing a flow of consciousness from which a dynamical *unity* and *identity* is achieved for oneself, others, and things.^29^ These embodied and enactive flows of consciousness frame the mental health problems in the life struggle *to be* as part of the individual’s ever-changing nature.

Fuchs and Pallagrosi continue: “*The primordial vulnerability of subjectivity is in other words an exposure to the other (Levinas 1991, 75, 112, 122; 1987, 146)*” [43, p. 12]. Levinas’ statement addresses a broad issue – namely, one’s own subjectivity is constantly exposed to otherness, thus being reciprocally co-constituted and influenced. In other words, *the human being continually faces the challenge of alterity, and therefore, mental health is understood as the capacity to continually develop an identity through this life-long process*. This process entails the constitution of *unity* and *identity* as a layered sense process culminating in human subjectivity in a continuous and co-constitutive relation with its environment and others. This process stems from the flow of consciousness.

Following M. Sheets-Johnstone, “…*an originary [sic] and even proper elucidation of how time-consciousness is possible lies not in the examination of something external to us, like a melody, but in the very nature of our being the animate organisms we are, that is in the very nature of self-movement*” [18, p. 35]. The point is that the constitutive flow of Ipseity and alterity is instantiated mainly through bodily movement as a continuous temporal and bodily movement flow between individuality and otherness; this continuous constitutive flow is embodied in the inter-*corporeal* dynamics (movement and action) and micro-dynamics (gaze, gesture, posture), and leads to a synchronic, fine-tuned inter-subjective dynamics, unveiling a shared inner-time consciousness.^30^

## Conclusion

This paper has looked to contribute a novel framework and approach to mental health, serving the theoretical and clinical realm. As stated above, the present work has argued for the following intuition: that bodily movement and temporality are fundamental psychotherapeutic assets for the development of conceptually guided approaches to mental health applicable for neuroscience, psychiatry and philosophy. Accordingly, the following phenomenological axes were proposed: *Temporal consciousness* (implicit, explicit) and *spatial consciousness* (*phenomenal field*, *life space*) [5]﻿, *bodily movement* (auto-affectivity, hetero-affectivity), *affectivity* (ante-predicative/predicative modalizations) and *intentionality* (ante-predicative/predicative modalizations). The latter variables are to be contrasted with other variables such as the genetic, molecular, cellular, physiological, behavioural, and dialogical-narrative; together, they assemble a mental health matrix for theoretical and/or clinical purposes. This conceptual work is grounded on the latter conceptual and phenomenological Husserlian tools.

To conclude, the proposed work is focused on a realm of subjectivity phenomena, which is correlated to the flow of consciousness and the constitutive process stemming from it. These phenomena and processes must be researched and studied to properly account for psychopathological disturbances. Accordingly, the present research offers a methodological challenge to the current conceptual and experimental work of science, which should better synthesize the phenomenological work articulated here with functional connectivity research on large scale brain networks.

There are three main advantages of the work proposed: (i) *economic*: as the patient grows in self-awareness and kinaesthetic *intelligence*,^31^ they are trained to notice alterations in their embodied experience which can anticipate crisis; they can also develop an organic embodied sense of healthy/unhealthy experiences; (ii) *therapeutic*: the phenomenological approach offers a rich ground from which new therapeutics can merge. As this approach is strongly based on the body, a sense of autonomy is promoted and integrated in the therapeutic approach. And (iii) *conceptual*: the present phenomenological perspective offers a ground and a solid foundation for mental health research to continue.

This account aims to promote a holistic conception of health and a positive conception of mental health by working through the continuum between personal development and psychopathological disturbances. Accordingly, this account looks to contribute to preventive work-ethos achievable for the general population through education and self-awareness. This work requires life-long educating on overcoming self-relation issues by enhancing self-awareness, developing skills for inter-corporeal resonance, attunement, and for building healthy strong relations, and finally, educating citizens so as to enable them to govern themselves within relations (social, political, economic) in an edifying and effective manner.

## Data Availability

Not applicable

## Footnotes

2 The *Otherness* which contributes to the constitution of selfhood [3, p. 338] is present in oneself (e.g. individual organic instinct-based reactions, but also in the presence of the other.

3 Physical culture is an overall health, aesthetics, pedagogy, and military training methodology rooted in classic Greek culture and originated during 19th century in Germany, UK, and US [7, p. 32., 8, p. 16].

4 Somatics is a body movement-based field of study and work, which promotes self-awareness [7, p. 16., 8, p. 16].

5 Francois Delsarte was a French oratory and singing trainer, dedicated to the study of the human body as an instrument of expression. He studied bodily movement and vocal behavior resulting in a system of expression [7, p. 38].

6 Pehr Henrik Ling was pioneer and founder of the Swedish physical education and gymnastics movement, which focused on calisthenics, breathing, stretching, and a particular type of massage [7, pp. 32–34., 8, pp. 24–25].

7 Refers to the concept used by Sheets-Johnstone [14, p. 21], signifying an organism that moves and experiences the spatial-temporal-energic dynamics of movement.

8 In a speculative manner, it could be suggested that this practical knowledge of embodied qualitative dynamics applies in a certain degree to the phylogenetic scale, between humans and other species with similar, dynamically structured experience and mirror neuron functional connectivity. This may suggest a phylogenetic epistemic capacity to sense and anticipate another’s embodied animated qualitative dynamics, in order to efficiently influence inter-subjective behaviours and reactions, as happens in everyday life.

9 Founded in 1988 by Jim Spira; at first located in the U.S. and then in Europe.

10 Based on Brian O’Shaughnessy’s *body image* [16] (understood as a primitive body structure grounded on kinaesthetic-proprioceptive dynamics and memory) and Maxine Sheets-Johnstone’s *tactile-kinaesthetic body* [18, pp. 26, 28], a bodily sense structure is proposed as a working hypothesis. The mechanical conditions of the articulated body and a sense of posture defines a repertoire of hierarchical possible movements, as a sense of the body’s functional structure. Additionally, through kinaesthetic-proprioceptive-tactile-affective dynamics [19, p. 12], synergies of meaningful movement are constituted, the likes of which if exercised in the long-run, *sediment an individual structured pattern of bodily movement dynamics*. This individual structured pattern becomes habitual through kinaesthetic memory [18, p. 44].

11 As speculatively commented in a past note, this ability applies to the individual’s own qualitative dynamics but also to another individual’s embodied dynamics, as a fundamental asset of a savvy social interaction.

12 Refers to a structured awareness in which individuals deal with object-like type of stimuli. For further information see Gallagher and Zahavi [20, pp. 56–57].

13 Refers to a propositional content of experience, adequate to truth-value consideration. For further information see: [21, chap. 6, 22 Lecture III].

14 These body-based therapeutic methodologies most often propose a movement-based organic and ecological approach to mental disturbances, which in the present work is briefly considered and elaborated upon in the conceptual realm. There is experimental evidence of considerable positive results that these therapeutic approaches have had, dealing with cases of severe [23] and mild [24] mental disturbances, enriching the scientific and clinical research to which the present work looks to contribute to from a conceptual ground.

15 Refers to the “*fundamental kinetic realities that inherently conjoin cognition and affectivity and make synergies of meaningful movement possible*” [19, p. 10].

16 According to the inter-subjective rhythm of reciprocal anticipation and actual feedback – of gesture, posture, movement, and andnarrative – as part of the inter-corporeal dynamics, the individual can sense how the other deals with alterity. This is part of the continuous social testing in which they can also make sense of the narratives displayed by each, as a complementary development of the inter-corporeal ‘proto-dialogue.’

17 Instantiated in habits, reactions, and moods as part of the otherness in oneself.

18 This concept is used laxly, as a complementary coincidence of the temporal dynamics (retention-protention) or synchrony between individual’s temporal experience.

19 According to Stanghellini [2, pp. 226 − 227], it is the alterity in the individual’s dispositions with which the individual cannot establish a dialectical relation.

20 According to Stanghellini [2, pp. 226 − 227], it is the alterity in the environmental events/circumstances with which the individual cannot establish a dialectical relation.

21 These spatial frames of reference are structured, the former through objective points of reference beyond the subject’s body (e.g. geographic parallels and meridians), and the latter through bodily subjective axis (which entails a direct and non-observational knowledge of the body), and therefore, a direct link between perception and action.

22 Progress in neuroscience could also contribute, but again it would require progress in conceptual *frameworks* rooted directly in embodiment.

23 Concept used laxly including any change in the individual condition.

24 As what is discussed are conceptual tools, in principle they serve academic and research purposes. As work continues to be done on developing psychopathological and psychotherapeutic approaches exploiting the virtues of these tools, a potential impact on mental health all-around can happen.

25 This is an ontological priority, which entails those individual narratives stem from individual embodied qualitative dynamics [35]. Notwithstanding, this issue is not the goal of this work.

26 The following proposal includes a complementary projected work. The theoretical is grounded in the study of the primal temporal constitutive and passive synthesis advanced by Husserl [36; 26; 27], which is proposed to be critically contrasted with the latest neuroscience work on Default Network [37], Neural correlates of temporality, and Default mode variability and temporal awareness. Default mode network (DMN) activity has been associated [38] with ‘mind-wandering’ (MW), conceived as an embodied meaningful experience. Definitions of MW have tangentially considered [39] the temporal structure of experience, although it has been a common denominator in experimental subject’s narratives. A phenomenological approach may define MW as a lived experience with an-other temporal dynamics. MW involves sensory-motor attenuation [40], also displayed in voluntary movement [41]; thus, an articulation of DMN’s activity, temporal dynamics of experience, and movement should be considered. Regarding the second component is the advancement of a phenomenologically-based therapeutic approach, which looks to enable individuals ‘to unfold their own, subjective and inter-subjective, time’ [42, p. 298]. A projected third component, to be conceived and developed with the latter, is a complementary experimental protocol yet to be designed.

27 Nonetheless, this is an issue to be studied and explored through embodied animated cognition.

28 This term is used to broadly characterize Parnas and Stephensen take of Kimura. A practical criterion to define the mentally healthy or unhealthy prompting condition is based on the ever-changing flow of consciousness and kinaesthetic dynamics, or its stationary condition, correspondingly.

29 The constitution of the dynamical unity and identity is a stratified process, both for animated bodies and things.

30 Based on the work of Krueger [47] and Laroche Berardi and Brangier [48, pp. 7–8], a sideline hypothesis is proposed: *‘We experience’ entails a shared inner-time consciousness*. Based on the following draft of an argument: (i) Interaction entails a mutual enaction, (ii) Mutual enaction entails a transparent incorporation of relational dynamics, (iii) Relational dynamics modulate affordances in the collective dynamics, (iv) Reciprocal collective dynamics entail a reciprocal pre-reflective background dynamics, (v) Reciprocal pre-reflective background dynamics entail a reciprocal inner-time consciousness dynamics, (vi) Collective dynamics entail coordinated reciprocal temporalities, (vii) Collective dynamics entail a reciprocally experienced inter-subjective time, and (Conclusion): ‘We experience’ entails a shared inner-time consciousness.

31 Understood as the practical knowledge of one’s bodily kinaesthetic (proprioceptive-tactile) dynamics, so that one could efficiently modify them autonomously [7, p. 76].
